# Evolution of long scalp hair in humans

**DOI:** 10.1093/bjd/ljae456

**Published:** 2025-01-22

**Authors:** Lo-Yu Chang, Maksim V Plikus, Nina G Jablonski, Sung-Jan Lin

**Affiliations:** School of Medicine, College of Medicine, National Taiwan University, Taipei, Taiwan; Department of Molecular Biology and Genetics, School of Medicine, Johns Hopkins University, Baltimore, MD, USA; Department of Developmental and Cell Biology, University of California Irvine, Irvine, CA, USA; Sue and Bill Gross Stem Cell Research Center, University of California Irvine, Irvine, CA, USA; Center for Complex Biological Systems, University of California Irvine, Irvine, CA, USA; NSF-Simons Center for Multiscale Cell Fate Research, University of California Irvine, Irvine, CA, USA; Department of Anthropology, The Pennsylvania State University, PA, USA; Department of Biomedical Engineering, College of Medicine and College of Engineering, National Taiwan University, Taipei, Taiwan; Department of Medical Research and Department of Dermatology, National Taiwan University Hospital, Taipei, Taiwan; Department of Dermatology, College of Medicine, National Taiwan University, Taipei, Taiwan; Research Center for Developmental Biology and Regenerative Medicine, National Taiwan University, Taipei, Taiwan

## Abstract

The ability to grow long scalp hair is a distinct human characteristic. It probably originally evolved to aid in cooling the sun-exposed head, although the genetic determinants of long hair are largely unknown. Despite ancestral variations in hair growth, long scalp hair is common to all extant human populations, which suggests its emergence before or concurrently with the emergence of anatomically modern humans (AMHs), approximately 300 000 years ago. Long scalp hair in AMHs was also a trait that was selected because it conveyed essential signals related to an individual’s age, sexual maturity, health and social status. Biologically, hair length is primarily determined by the amount of time that a hair follicle spends in the active growth phase (anagen). While anagen duration is typically tightly regulated in most mammals, the inherent ability of a hair follicle to continuously recruit new dividing progenitors to its base, where hair fibre is generated, theoretically removes limits on maximal anagen duration. We propose a model wherein hair cycle progression into and out of anagen is regulated by evolutionary malleable molecular checkpoints. Several animal species and domesticated animal breeds display long body hair, which suggests that extremely long scalp hair in humans emerged via attenuation of an existing out-of-anagen checkpoint mechanism rather than via a newly evolved molecular programme. Studying congenital and somatic mosaicism conditions featuring altered hair length could potentially unveil the currently unknown molecular basis underlying this human trait.

## Teaser

Long scalp hair is likely to have evolved first as long, tightly curled hair for better thermoregulation and was further selected at a later point in human evolution owing to its unique social communication functions. An extremely long growth period of scalp hair could be the result of the attenuation of an out-of-anagen checkpoint mechanism. Studying congenital conditions characterized by altered hair length may potentially shed light on the molecular basis for this human-specific trait.

## Long scalp hair: benefits and disadvantages

**Figure 1:**
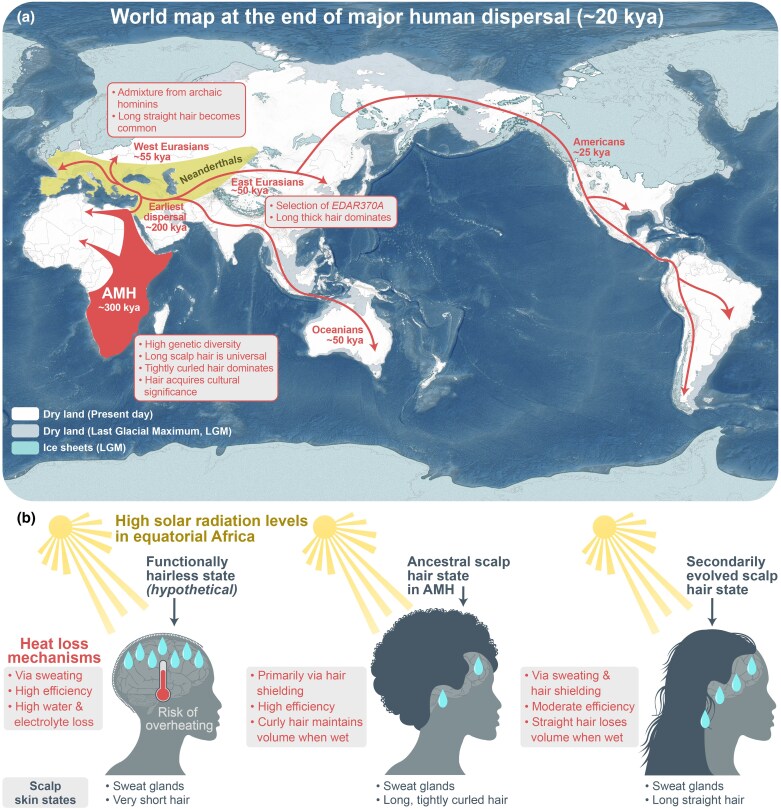
Evolution of scalp hair traits in anatomically modern humans (AMHs). (a) AMHs evolved in Africa approximately 300 thousand years ago (kya). African AMH populations were genetically diverse and broadly featured long, tightly curled scalp hair, which was styled for visual communication. Upon dispersal from Africa starting approximately 200 kya (red arrows), AMHs had contact with and experienced genetic admixture from archaic humans (the area showing the Neanderthals’ range is shaded in yellow). From Western Asia, humans dispersed into Europe, Oceania, Central and Eastern Asia, and then into North and South America (around 25 kya). Long scalp hair remained a universal anatomical feature in all non-African populations, while hair curliness and thickness further diversified. Dispersal history of AMHs is adopted from Nielsen *et al*.^93^ Further information on the timeline of hominin evolution is provided in Figure S1 (see Supporting Information).^94–96^ The background image represents an approximate world map during Last Glacial Maximum (LGM), approximately 20 kya. (b) The proposed primary role of long, tightly curled scalp hair in AMHs. The Equatorial Africa region represented the original ecological niche of AMHs and was characterized by high-intensity solar radiation. The human head was exposed to direct sunlight, posing a risk of overheating which could affect the brain. To counter overheating, increased sweat gland density enabled active heat loss via perspiration. However, perspiration results in significant loss of water and electrolytes (left). Evolution of long scalp hair enabled physical head shielding, reducing direct sunlight exposure of the skin and the required amount of perspiration (centre and right). However, long straight hair is prone to losing volume when wet, which decreases its head-shielding capacity (right), whereas long, tightly curled hair has better water resistance and maintains high volume, thus offering robust head-shielding efficacy (centre). Long, tightly curled hair evolved as the ancestral scalp hair state in AMHs.

Hair is a signature mammalian characteristic with versatile functions, including thermoregulation, protection from ultraviolet radiation, physical and chemical insults, sensation of pain, vibration and touch, and defence from predators.^1–3^ Human hair patterns, which feature prominently reduced body hair length combined with extremely long scalp hair,^4–8^ are an outlier among mammals. The likely original function of long scalp hair was to shield the sun-exposed head of upright-standing human ancestors. Long scalp hair probably reduced the amount of sweat secretion required to counter the total thermal load experienced by individuals from incoming solar radiation in equatorial Africa, and from endogenous muscle-generated heat during exercise.^9^ Tightly curled scalp hair is more efficacious at reducing heat gains compared with other hair shapes, and such hair probably represents the ancestral scalp hair form (Figure 1).^9^ Variability in hair shapes increased over time. These variations are thought to be associated with the dispersal of anatomically modern humans (AMHs) and accompanying effects of populational bottlenecks, admixture with Neanderthals and Denisovans, and adaptations to diverse environments at new geographic locations (Figure 1a).^10^ Extreme scalp hair length was probably universal across all African AMH populations and available for diverse functions, other than thermoregulation. In this sense, long scalp hair is an excellent example of exaptation, a form of evolutionary co-option,^11^ whereby it acquired secondary essential functions in communicating social cues.^12^ Under these conditions, unwanted hair loss triggered significant psychological stress in affected individuals.

Despite variations, both across different species and different body regions, hair typically has a finite length. A fully grown hair fibre commonly remains attached within its hair follicle (HF) until a new round of growth replaces it. In a typical adult human, approximately 90% of scalp HFs are in active growth (anagen) at any given time, which lasts for 5–7 years.^13,14^ This is in contrast to small (vellus) body HFs, which have short-lasting anagen, such as 22–28 days on the upper arm.^15^ When large (terminal) scalp HFs reduce in size, start growing vellus-like hairs, and/or stop growing for an extended period, they are considered to be entering a pathological state.

Elucidation of long scalp hair roles in human prehistory requires further investigation of its thermoregulatory benefit vs. the physical burden it may have caused by hindering vision and locomotion. Continuous hair growth is also metabolically expensive, requiring synthesis of large quantities of keratins and keratin-associated proteins.^1,2,16,17^ Conversely, highly visible long scalp hair effectively communicates a good state of fitness, whereas compromised hair growth implies poor nutrition and disease.^17,18^ Indeed, kwashiorkor, a disease caused by severe dietary protein deficit, features dramatic hair thinning and depigmentation.^19^ The ornamental potential of long hair enables the use of distinct hairstyles to signify a person’s social position, creativity and manual skills.^20^ Therefore, in prehistory, hair styling likely became an essential part of social communication, which probably further promoted long-hair trait selection.

## Hair evolution: evidence from prehistory

In nonhuman primates, including chimpanzees (*Pan troglodytes*), hair is mostly straight. Hair curliness is a distinct human characteristic, yet whether it has evolved before, after or in parallel with scalp hair elongation remains unknown. Tight curliness contributes to higher scalp hair volume, which in turn increases its thermoregulatory capacity (Figure 1b).^21^ However, curly hair is more prone to breakage from wear and tear,^22^ which may have facilitated selection of extremely long hair growth as a compensatory trait. Thus, evolution of hair curliness may have preceded evolution of extreme hair length in AMHs or their hominin ancestors. Self-styling ability of tightly curled hair, which partially relieved drawbacks of excessive hair length, might have facilitated coevolution of both hair traits.

**Figure 2:**
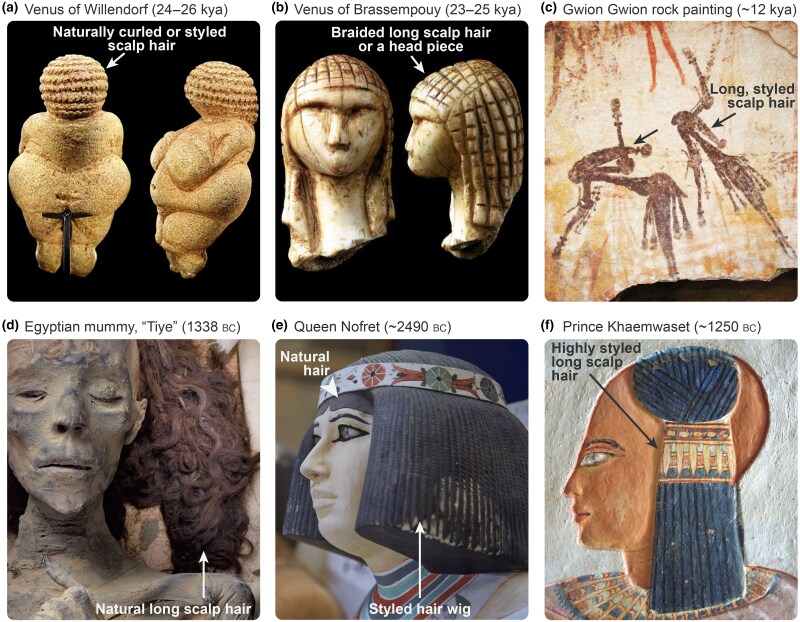
Long scalp hair and hairpieces in prehistoric art and during early history. (a) Venus of Willendorf from modern day Austria dating from approximately 24–26 thousand years ago (kya). The head features prominent covering, which may represent either natural curly hair or a hairpiece (arrow). (b) Venus of Brassempouy from modern day France dating from approximately 23–25 kya. The head features prominent covering, which might represent braided long scalp hair or a headpiece (arrow). (c) Gwion Gwion rock painting from modern day Western Australia dating from approximately 12 kya. It depicts two human figures with likely long, styled scalp hair (arrows). (d) Egyptian mummy of Tiye, the great royal wife of pharaoh Amenhotep III (18th dynasty; year of death 1338 Bc). It features natural long, wavy scalp hair (arrow). (e) Princess Nofret from the fourth dynasty of Ancient Egypt (approximately 2490 Bc). The head is covered with a styled wig (arrow) placed over natural hair, which is visible above the forehead and straight in appearance (arrowhead). (f) Painted bas-relief from tomb QV44, depicting prince Khaemwaset, son of pharaoh Rameses III (20th dynasty; approximately 1250 Bc). The head features a hairstyle typical of children in Ancient Egypt at that time, namely long, braided hair on a side and shaved surrounding skin.

AMHs emerged in Africa approximately 300 thousand years ago (kya) (Figure S1; see Supporting Information).^23^ Relatively small human groups probably dispersed from Africa as early as 200 kya,^24^ eventually disseminating worldwide as diverse non-African populations (Figure 1a). The genetic bottleneck effects that dispersing small bands of humans experienced as they encountered geographic barriers resulted in reduced genomic variation among modern non-African populations compared with modern African populations.^25,26^ The timing and genetic bases of diverse scalp hair forms, including straight hair, among dispersing AMH populations remain unclear. Yet, depictions of people with long scalp hair and elaborate hairstyles across Palaeolithic caves, rock paintings and carved figurines suggest that long hair capable of being styled was universal across all prehistoric populations. ‘Venus figurines’ from Europe from the Upper Palaeolithic era (approximately 28–22 kya) feature heads adorned with scalp hair, including long to shoulder-length hair (Figure 2a, b), although some historians maintain that these figurines depict hairpieces.^27^ Western Australian ‘*Gwion Gwion*’ rock art from approximately 12 kya depicts humans wearing cone-shaped headwear or long styled hair (Figure 2c).^28^ In early history, long hair styling became more elaborate. Ancient Egyptians wore diverse hairstyles made of both natural and false hair, which were decided according to social and political norms to convey an individual’s status (Figure 2d–f).^20,29,30^ Long scalp hair was found in mummies (Figure 2d) and, for example, Ancient Egyptian children wore long, braided hair on one side with the rest of the scalp cleanly shaved (Figure 2f).

## Scalp hair characteristics of modern humans

There is a diverse range of hair morphologies and growth profiles among AMHs.^31^ Hrdy first systemically categorized scalp hair forms using eight variables, including hair diameter, medullation and curvature.^32^ Recent comparison of hair density, growth rate, diameter, colour, curliness and telogen (rest state) vs. anagen HF frequencies among 2249 young adults of 24 ancestral origins across five continents showed that each parameter changes continuously, with a high degree of overlap.^31^ Thus, genetic determination of scalp hair growth is likely to be multifactorial, analogous to body height determination.^33^ For example, scalp hair density varies between 153 and 208 hairs per cm^2^, whereas hair growth rate ranges between 272 and 427 µm per day.^34^ Consistent with higher genetic diversity among African populations, these populations also display a high diversity of scalp hair growth profiles.^34^ Populations outside of sub-Saharan Africa show increased scalp hair growth rates and densities,^31^ which could be changes possibly caused by genetic admixture from Neanderthals and Denisovans, although recent genomic analysis suggests that the impact of archaic hominin DNA on physical attributes of AMH skin is minimal.^35^ The inner root sheath, a cylindrical cell layer that hardens ahead of hair fibre, is thought to ‘guide’ originally symmetric fibre into asymmetric shapes, such as curly shapes.^36^ Concordantly, curly hair shapes are linked to polymorphism in genes encoding keratin, trichohyalin and copper transporter protein expressed by inner root sheath cells.^36^ Conversely, the *EDAR370A* variant of the ectodysplasin-A receptor gene correlates with straight and thick scalp hair in modern East Asian people, and it probably originated in the region of modern central China approximately 30 kya.^37^ Because the earliest inhabitants of the Americas were of Asian descent, straight scalp hair found in 9000-year-old natural mummies in Chile was attributed to a likely *EDAR370A* variant.^38^

## Hair follicle morphology and growth cycle

**Figure 3:**
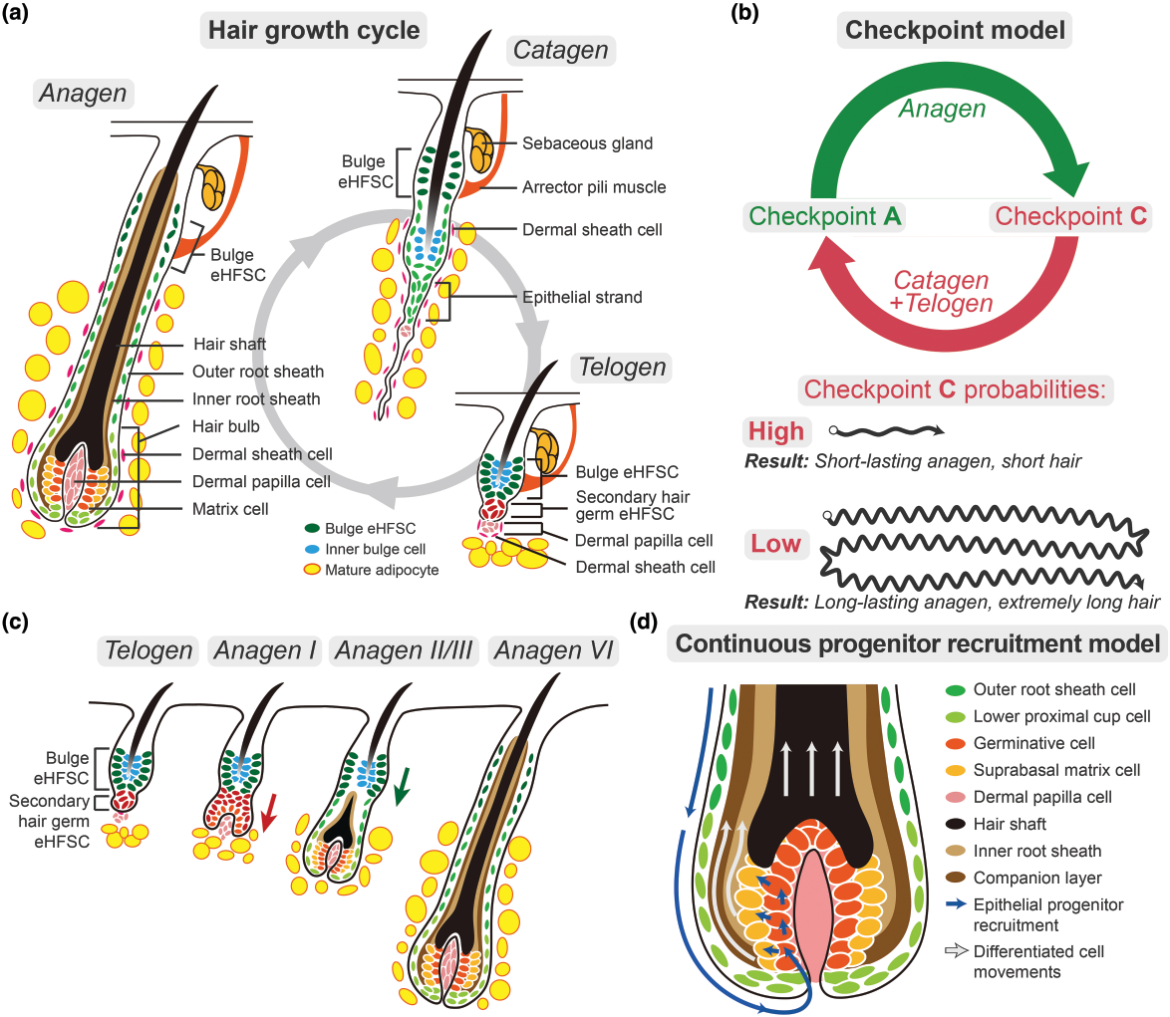
Hair follicle morphology, cellular dynamics and key regulatory events across hair growth cycle. (a) Schematic drawing of the hair growth cycle. Bulge and secondary hair germ epithelial hair follicle (HF) stem cells (eHFSCs) are quiescent during telogen. They transiently activate during anagen onset to regenerate the lower HF segment. During the catagen phase, the lower HF segment regresses, driven by apoptosis. Key HF cell types and compartments are annotated and colour-coded. (b) Hair growth cycle checkpoint model. The growth cycle can be conceptualized into two phases, anagen (green) and catagen/telogen (red). The progression of the HF through these phases is controlled at checkpoints C and A, which regulate transition from anagen to catagen/telogen and from catagen/telogen to anagen, respectively. The lower portion of the figure depicts hypothetical results of hair growth under high and low checkpoint C probabilities. The results are depicted as vectors, whose length represents resulting hair fibre (short for high checkpoint C probability and extremely long for low probability). (c) Cellular dynamics of eHFSCs upon anagen initiation and progression. Primed secondary hair germ eHFSCs activate during anagen subphase I, while bulge eHFSCs activate with a delay during anagen II/III. eHFSCs proliferate rarely during other phases. (d) Cellular dynamics in the anagen HF bulb under continuous progenitor recruitment model. Germinative matrix cells proliferate and subsequently differentiate into trichocytes (short blue arrows) to support continuous hair elongation and that of associated sheaths (long white arrows). Outer root sheath and lower proximal cup cells have progenitor properties and can continuously migrate towards the hair matrix to, in principle, replenish ‘exhausted’ germinative cells. This ‘resupply’ mechanism (long blue arrow) endows anagen HFs with the potential for an extended growth.

The HF is a skin miniorgan that periodically cycles through three consecutive phases of growth, i.e. anagen, catagen (involution) and telogen (Figure 3a).^39,40^ The HF consists of the upper permanent compartments including the bulge, and lower transient compartments that reside below the bulge and remodel throughout the growth cycle.^39,40^ The bulge of both telogen and anagen HFs harbours quiescent epithelial HF stem cells (eHFSCs). During telogen they reside close to dermal papilla fibroblasts (DPFs), which secrete inhibitory signals that promote eHFSC quiescence. When anagen starts, DPFs switch to making activating signals.^41,42^ Consequently, eHFSCs respond by proliferating and generating the epithelial lineage of the actively growing the HF, including germinative cells in the hair matrix (Figure 3a, c).^2,43^ Highly proliferative matrix cells produce terminally differentiated trichocytes that assemble into a hardened hair fibre and surrounding inner root sheath. Thus, the continuous supply of and proliferation by matrix cells are essential for continuous hair growth (Figure 3a). However, normally after a certain period of time, anagen HFs initiate an involution programme. This terminates matrix cell proliferation, stops hair growth and triggers apoptotic regression of most of the catagen HF epithelium, a process that remodels HF back to its dormant state (Figure 3a).^2^

## Continuous progenitor recruitment removes limits on anagen duration

The maximal hair length is primarily determined by the duration of anagen and, secondarily, by the rate at which fibre grows. An anagen phase in human scalp HFs that lasts 5–7 years translates to hair that is approximately 50–110 cm long.^13,14,31^ Guinness World Records certified the longest known human scalp hair at 5.627 m in a 44-year-old Han Chinese woman.^44^ While this example represents an exceptional case, it illustrates the natural growth potential of a human scalp HF.

At the tissue level, the maximal possible anagen duration is limited by the total proliferative potential of hair matrix progenitors, which display features of short-lasting transit amplifying cells.^43,45^ In human HFs, matrix cells divide approximately once per day.^2^ Hypothetically, to produce a hair that is 4 m long would require continuous anagen for 30 years and around 11 000 division rounds by each matrix cell. It is challenging to reconcile such a mitosis-heavy process with the naturally low proliferative capacity of matrix cells, assuming their population is finite. However, extremely long-lasting hair growth can be solved with the assumption that hair matrix continuously recruits additional proliferative progenitors from adjacent HF compartments. Indeed, a seminal lineage tracing study on mouse vibrissa HFs, which exhibit relatively long-lasting anagen, showed that eHFSC progeny continuously migrate downward out of the bulge, along the so-called outer root sheath and towards the hair bulb.^46^ Moreover, epithelial cells in the outer root sheath and its lowermost segment called the lower proximal cup, can migrate into the hair matrix where, in principle, they can replace ‘ageing’ germinative cells that have reached proliferative exhaustion either naturally or following genotoxic injury (Figure 3d).^47,48^ Thus, theoretically, very long hair can be enabled by eHFSCs or outer root sheath progenitors continuously resupplying matrix. In this framework, anagen termination is not a ‘passive’ inevitable result of proliferative matrix exhaustion, but rather it is an actively controlled molecular process.

## Hair growth regulation by cycle checkpoints

**Figure 4:**
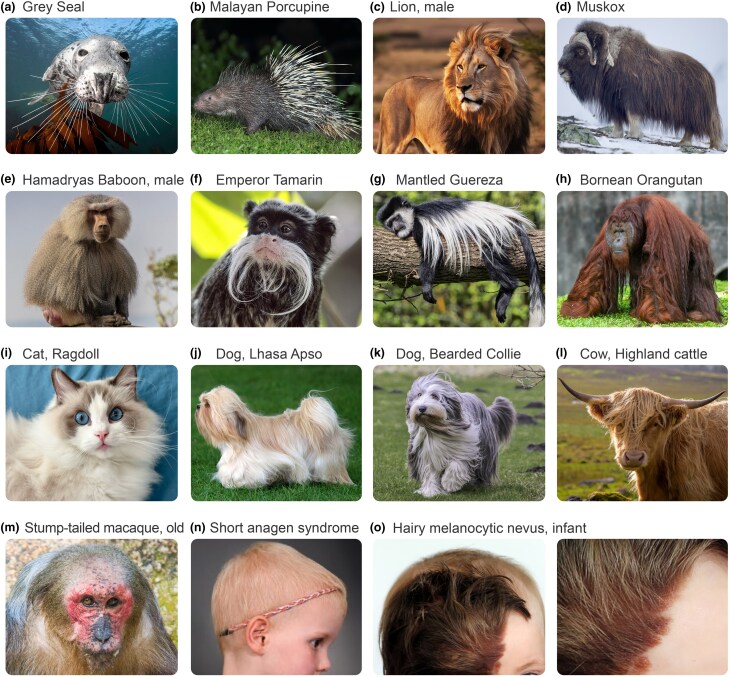
Hair length flexibility in animals and in humans upon conditions with altered hair growth. (a–h) Examples of animal species with naturally occurring long hair. (a) Grey seals (*Halichoerus grypus*) feature long vibrissa hairs that form a sensory ‘sphere’ around the snout. (b) Malayan porcupines (*Hystrix brachyura*) feature very long dorsal quill hairs. (c) Adult male lions (*Panthera leo*) feature long mane hairs. (d) Muskoxes (*Ovibos moschatus*) grow long pelage hair, which drapes over the body. (e–h) Examples of primates with long hair. (e) Adult male Hamadryas baboons (*Papio hamadryas*) grow long facial and body hair. (f) Emperor tamarins (*Saguinus imperator*) grow long facial hair. (g) Mantled guerezas (*Colobus guereza*) grow distinctly coloured long dorsal body hair. (h) Adult male Bornean orangutans (*Pongo pygmaeus*) grow very long body hair, especially in captivity. (i–l) Examples of domesticated animal breeds with long hair: (i) Ragdoll cat (*Felis catus*), (j) Lhasa Apso and (k) Bearded Collie dogs (*Canis lupus familiaris*), and (l) Highland cattle (*Bos taurus*). (m) Head of an old stump-tailed macaque (*Macaca arctoides*) displaying frontal hair loss. (n) Example of short anagen syndrome in patient carrying heterozygous *WNT10A* variant. Scalp hair is sparse and short, frontal hairline is high. (o) Example of a hairy melanocytic naevus featuring long, thick and darkly pigmented hair.

Consistent with the above theory, empirical evidence supports the idea that there is a precise anagen termination mechanism. Within the same species, HFs exhibit both flexibility and precision of hair growth duration. Many mammals feature short pelage hairs and long vibrissa hairs, and the length of these hairs is proportional to anagen durations that the respective HFs can sustain. For example, in mice (*Mus musculus*), pelage hairs grow for approximately 16 days, whereas vibrissa hairs can grow for over 2 months.^49^ Intriguingly, among individual vibrissae in the same animal, such as the grey seal (*Halichoerus grypus*), there is significant length gradation. By forming an orderly array, long and short vibrissae produce a sphere of hair tips around the animal’s snout (Figure 4a). Numerous phylogenetically diverse species can grow very long pelage hair (Figure 4b–h). For example, in adult male lions (*Panthera leo*), mane hair can grow up to 20 cm long (Figure 4c).^50^ Extinct Woolly mammoths (*Mammuthus primigenius*) grew very long body hair, with the longest hairs, that draped from the body like a skirt, reaching up to 90 cm.^51^ An analogous long hair ‘skirt’ also forms in muskoxes (*Ovibos moschatus*) (Figure 4d). Among primates, long pelage hair can grow in hamadryas baboons (*Papio hamadryas*), emperor tamarins (*Saguinus imperator*), mantled guerezas (*Colobus guereza*) and orangutans (*Pongo pygmaeus*), for example (Figure 4e–h). Male adult orangutans, especially those living in captivity, can grow hair longer than 1 m (Figure 4 h). Furthermore, certain breeds of domesticated animals, including cats, dogs and cattle, can grow long body hair (Figure 4il). Therefore, the potential for long hair growth is conserved and recurrent among mammals.^52^

Given that catagen duration is relatively constant and that catagen is necessarily followed by telogen, the cyclical process of hair growth can be conceptually simplified into two phases: anagen and catagen/telogen. Analagous to the cell cycle, we propose that the hair growth cycle is controlled at two checkpoints, i.e. checkpoint A for anagen entry and checkpoint C for anagen exit (Figure 3b).^53^ Thus, hair length can be modulated by enhancing or suppressing the passing of the anagen HF through checkpoint C. Indeed, the HF’s entry into and out of anagen can be prominently modulated by a variety of molecular signals, including hormones, cytokines and growth factors,^2,41,54–59^ whereas prolonged arrest in the telogen phase and, thus, inability to pass through checkpoint A, is an essential pathophysiological manifestation of both androgenetic alopecia and alopecia areata, two hair loss conditions featuring significant changes in local signalling mileu.^2,60^ The HF’s ability to respond to numerous catagen/telogen inducers and suppressors allows for flexible control over anagen duration. Although the exact physiological inducers of checkpoint C remain undetermined, they may include growth factors that form part of the fibroblast growth factor (FGF), epidermal growth factor (EGF) and transforming growth factor (TGF)-β families of molecules, some of which have been shown to promote catagen.^55,61,62^ The exceptional anagen lengthening in human scalp HFs can be caused by higher levels of a suppressor (or suppressors) of such an inducer (or inducers) and the resultant low probability of exit through checkpoint C. Consequently, by default, anagen in scalp HFs persists without a definite duration. This is in contrast to HFs in other body sites, which show relatively fixed anagen durations. Growth cycle timing in each adult scalp HF is not synchronized with its neighbours, and catagen entry appears to be stochastic.^13^ However, it is notable that scalp HFs efficiently execute checkpoint C when presented with supraphysiological inducers, such as stress, seasonal changes and genotoxic damage. In these scenarios, many anagen HFs synchronously enter catagen,^2,63,64^ resulting in a dramatic hair loss. This is particularly obvious in patients with cancer on chemotherapy or radiation therapy regimens.^48,65,66^

## Regionally specific regulation of scalp hair

Localized growth of extremely long hair on the scalp implies the existence of a scalp-specific molecular programme; however, how and when hominins acquired such a programme remains unknown. Androgenetic alopecia is a common syndrome characterized by progressive terminal-to-vellus transformation of scalp hair.^67^ Mechanistically, 5-α-reductase activity in DPFs increases local production of dihydrotestosterone, a more potent form of testosterone.^67^ Long-term exposure to elevated dihydrotestosterone alters DPFs, compromising their expression of growth factors essential to sustain long-lasting anagen.^60,67^ Patterned scalp hair loss is not unique to humans, and an androgenetic alopecia-like state also occurs in nonhuman primates; however, the similarity to human hair loss is disputed.^67,68^ In stump-tailed macaques (*Macaca ­arctoides*), male macaques generally have bald foreheads upon reaching adulthood (Figure 4 m).^68^ Members of the *Hominidae* family, including chimpanzees, gorillas (*Gorilla gorilla*) and orangutans, also show hair loss patterns that resemble androgenetic alopecia.^68^ These examples highlight that the gene regulatory network for scalp-specific hair growth and hair loss already existed in nonhuman primates. Theoretically, further alterations in this network in pre-AMH hominins could have produced low-efficiency checkpoint C, enabling extremely long-lasting anagen and, consequently, extremely long scalp hair.

Prominent hair growth regionalization in the human scalp implies body site-specific expression of growth cycle regulators.^69^ Regionalized molecular programmes are commonly driven by patterned heterogeneity of the mesenchyme,^70,71^ which is controlled by homeobox (HOX) genes, transcriptional factors regulating establishment of body axes and segments.^70,72^ In the skin, patterned HOX activity is prominent and accounts for regional differences such as expression of specific keratin genes in thickened glabrous skin epidermis.^73^ Patterned HOX activity is regulated epigenetically and becomes fixed after morphogenesis.^74^ Indeed, skin and hair properties are maintained when parts of the skin or individual HFs are transplanted to new body sites.^75^ Within HFs, HOX gene expression is prominent in DPFs, which regulate eHFSC activity and anagen duration.^76^ In mice, high expression of *Hoxc* genes by DPFs promotes Wnt signalling that facilitates eHFSC activation, leading to frequent hair regrowth and higher fur density. As DPFs also determine hair fibre diameter and structural features by modulating differentiation of matrix cells,^77,78^ DPFs may also ‘store’ molecular information on anagen duration.^2,79^ Therefore, scalp hair elongation in humans could be mediated by an epigenetic regulatory process affecting the secretome of DPFs. Consistent with the essential role of DPFs in hair growth, cell therapy for alopecia using cultured DPFs or related dermal sheath cup fibroblasts has been proposed;^80^ however, it remains unknown whether DPFs can stably maintain molecular features that determine hair morphology and/or anagen duration in a prolonged culture.^81^

## Learning about the molecular basis of long scalp hair from human dermatological conditions

Studying human dermatological conditions that involve altered hair length might provide clues about the molecular basis for long scalp hair. In familial trichomegaly, caused by loss-of-function mutations in *FGF5*, body hair and eyelashes become excessively long owing to a prolonged anagen phase.^54^ Similarly, a prolonged anagen phase is also observed in *Fgf5*-mutant domesticated animal breeds, such as particular breeds of cats^82^ and dogs^83^ (Figure 4i–k). FGF5 signals to DPFs in anagen HFs,^84^ promoting catagen entry. Yet, because germline *FGF5* mutations prolong anagen over the entire body, potential modifications of this candidate gene for long scalp hair might lie in its regulatory element(s) rather than its protein coding sequence.

Conversely, patients with short anagen syndrome (Figure 4n) feature abrogated anagen duration in scalp HFs without other ectodermal dysplasias.^85^ In these patients, body hair is generally normal, which suggests the lack of any single gene mutation that causes generalized HF dysfunction and, rather, pointing to location-specific dysregulation in hair cycle regulators. Short anagen syndrome is associated with *WNT10A* variants and, intriguingly, mice null for *Wnt10a* show premature catagen entry and delayed anagen induction.^85^ Notably, changes in *WNT10A* expression have also been implicated in androgenetic alopecia.^85^

Molecular insights can also be obtained by studying conditions that involve localized hypertrichosis, such as hairy melanocytic naevi. The latter are caused by accumulation of senescent melanocytes and can occur on any part of the body, where normally vellus HFs convert into a terminal state and grow uncharacteristically long hair. A recent study showed that senescent melanocytes secrete osteopontin, a potent eHFSC-activating molecule normally produced by DPFs.^86^

We propose that the evolutionary change responsible for an extremely long scalp hair growth is probably not a loss- or gain-of-function mutation (or mutations) in any given protein coding gene, but rather a change (or changes) in the broader genomic structure, such as in regionally restricted enhancers or a rearrangement event affecting a Hox-dependent gene network, with these changes, in turn, altering the expression of locally acting hair-cycle modulating factor(s). Furthermore, because genomic effects are commonly pleiotropic, several phenotypes may result from such changes, with only some phenotypes being clearly beneficial. For instance, in relation to the aforementioned *EDARV370A* variant attributed to thick scalp hair in East Asian populations, the primary adaptive effects may have been increased mammary gland branching and/or eccrine gland density rather than hair changes.^87,88^ A similar pleiotropic scenario may have also accompanied the evolution of long scalp hair in hominins, whereas prolonged anagen was at first a ‘side-­effect’ hitchhiking on another adaptive trait.

## Conclusions

The evolution of extremely long scalp hair has significantly added to the social communication repertoire in modern humans. Extreme elongation of scalp hair during evolution probably stems from the release of a natural molecular brake on anagen termination, which allows scalp HFs to grow continuously for years. While the evolutionary history, genetic basis and precise molecular mechanisms that produce the long scalp hair trait remain poorly understood, in this review we have summarized its probable adaptive benefits and disadvantages, and discussed the existence of regionally specific control and trait-driving candidate genomic alterations. We also raised key open questions regarding this paradoxical hair growth feature of humans.

In light of current advances in the methodologies that allow human tissues to be studied, the time is ripe for starting to dissect the molecular basis of this enigmatic human hair trait. In particular, we anticipate that deeper molecular insights into human hair growth regulation will come from profiling vellus body HFs vs. terminal scalp HFs vs. androgenetic alopecia-affected miniaturized scalp HFs using single-cell RNA sequencing and next-generation spatial sequencing technologies. These efforts have already begun,^89,90^ but large-scale studies on individuals of diverse ancestries will be essential to improving our understanding of this trait. Moreover, advances in organotypic human HF culture,^91^ skin organoid culture^92^ and human-on-mouse HF xenograft models^40,42,86^ are currently enabling validation of candidate regulators of long-lasting anagen in addition to preclinical testing of hair growth-enhancing drug candidates.

## Supplementary Material

ljae456_Supplementary_Data

## Data Availability

No new data were generated or analysed in support of this research.
