# Supplementary material for: Evolution of long scalp hair in humans
Source: Br J Dermatol. 2025 Jan 22;192(4):574–84. doi: 10.1093/bjd/ljae456 (PMC11918595; doi:10.1093/bjd/ljae456)
Supplement: ljae456_Supplementary_Data [file ljae456_supplementary_data.pdf]

Supplementary Figure

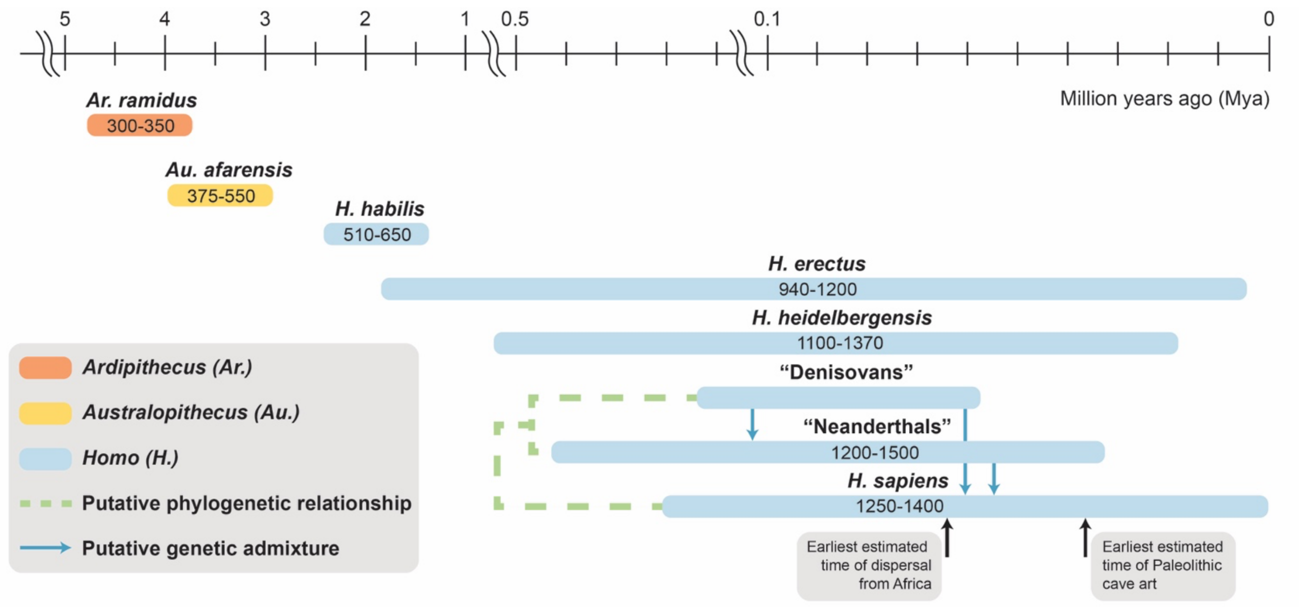

Supplementary Figure 1. A simplified timeline of hominin evolution[23,95-97]. Graph depicts timeline of major hominin species based on fossil and genetic evidence. Estimated brain capacity for each species is indicated within associated colored bar in cubic centimeters. Exact timing of the emergence of long scalp hair trait in hominins remains unknown due to the lack of direct fossil evidence. *Ardipithecus ramidus* was facultative bipedal species. Facultative bipedalism and later obligate bipedalism might have been a trait in early hominins that contributed to the retention of the long scalp hair trait by allowing more hand freedom to manage scalp hair. Higher intelligence, sophisticated hand control to trim hair and better hunting skills conferred by larger brain capacity could also have helped early hominins to preserve the long scalp hair trait.
